# The use of 2% chlorhexidine gluconate in 70% isopropyl alcohol for skin disinfection prior to central venous catheterization in infants: a national survey of the Italian Society of Neonatology

**DOI:** 10.1186/s13052-025-02016-5

**Published:** 2025-06-06

**Authors:** Vito D’Andrea, Antonella Capasso, Carmen Rodriguez-Perez, Francesca Tota, Rossella Mastroianni, Giorgia Prontera, Giovanni Barone

**Affiliations:** 1 Neonatology Unit, Department of Woman and Child Health and Public Health, Fondazione Policlinico Universitario “Agostino Gemelli” IRCCS, Largo Agostino Gemelli N 8, 00168 Rome, Italy; 2 Neonatology and Neonatal Intensive Care Unit, A.O.U. Federico 2, Naples, Italy; 3 Neonatology and Neonatal Intensive Care Unit, ASST Spedali Civili, Ospedale Dei Bambini, Brescia, Italy; 4 Neonatal Intensive Care Unit, Ospedale S. Chiara, APSS, Trento, Italy; 5 Neonatal Intensive Care Unit, S. Anna E S. Sebastiano Hospital, Caserta, Italy; 6 Neonatal Intensive Care Unit, Infermi Hospital, Rimini, AUSL Romagna, Rimini, Italy

**Keywords:** Skin disinfection, Chlorhexidine gluconate, Central line bloodstream infections, Premature infants

## Abstract

**Background:**

2% Chlorhexidine gluconate in a 70% alcohol solution is considered the gold standard for skin disinfection in adult and paediatric patients prior to insertion of a central venous catheter. The use of chlorhexidine gluconate in infants remains controversial due to concerns about potential risks of skin injury such as chemical burns, contact dermatitis and systemic absorption. The objective of this survey is to investigate the practice of skin disinfection with 2% chlorhexidine gluconate in 70% isopropyl alcohol prior to central venous catheterization in infants in Italy.

**Methods:**

Over a one-year period, we conducted an online survey in 114 neonatal intensive care units on the use of skin antiseptics for central venous catheters in neonates.

**Results:**

Sixty-five percent of the centers use a 2% chlorhexidine solution in a 70% alcohol solution for skin disinfection before inserting central catheters in neonates. Ten percent use povidone-iodine, and two percent use sodium hypochlorite. Among those using alcoholic chlorhexidine, sixty-five percent use it without any restrictions, thirty percent limit its use based on the gestational age of the neonate, and five percent limit it based on the neonatal weight. Thirty-seven percent of the centers rinse off the chlorhexidine solution due to concerns about chemical injuries.

**Conclusions:**

The survey revealed that there’s still a lack of uniformity in the use of skin disinfectants before central catheter placement. The Neonatal Vascular Access Group (AVN GdS) of the Italian Society of Neonatology reiterates the use of 2% chlorhexidine in a 70% alcohol solution for skin disinfection before central catheter insertion, as recommended by recent guidelines. However, specific precautions must be taken to avoid complications.

**Supplementary Information:**

The online version contains supplementary material available at 10.1186/s13052-025-02016-5.

## Background

Skin disinfection is a crucial and essential component of catheter insertion and maintenance bundles. However, there’s a lack of clear evidence regarding the optimal solution for disinfecting neonatal skin, making it a controversial topic [1].

Varying combinations of alcohol and povidone-iodine have been used in the Neonatal Intensive Care Unit (NICU). Chlorhexidine gluconate (CHG) is increasingly being used in the NICU. Surveys of neonatal practices show a shifting trend in skin disinfection, with a substantial number of NICUs incorporating CHG as part of their skin disinfection protocols [2].

This growing trend is primarily driven by concerns about the effectiveness of povidone-iodine and its potential inhibitory effects on the thyroid gland of premature infants [3]. Additionally, in pediatric and adult populations, CHG in an alcoholic solution is strongly recommended for its efficacy and safety profile [4].

Despite the increasing use of CHG in neonatal intensive care units (NICUs), there are limited recommendations to guide its safe use in this vulnerable population. While the Centers for Disease Control and Prevention (CDC) recommends CHG as part of skin disinfection to prevent central line bloodstream infections (CLABSIs), it has not made any specific recommendations regarding the safety or efficacy of CHG use in infants under two months of age [5]. The Food and Drug Administration (FDA) safety information for CHG cautions “use with care for premature infants or infants under 2 months of age” [6]. The most recent neonatal skin care clinical practice guideline from the Association of Women’s Health, Obstetric and Neonatal Nurses (AWHONN) [7] cites “insufficient evidence to recommend a single product for all neonates and procedures” in relation to skin disinfectants.

Considering the high level of uncertainty from multiple organizations and the significant impact that the systematic use of CHG in 70% isoprophyl alcohol would have as a strategy to prevent CLABSI, the Neonatal Vascular Access Group (AVN GdS) of the Italian Society of Neonatology believed that it was worthwhile to survey neonatal practice on skin antisepsis in the Italian NICU.

## Methods

A prospective cross-sectional survey study was conducted from November 2022 to November 2023 among all 114 Italian Neonatal Units. A survey comprising 18 questions was developed utilizing a modified Delphi method [8]. Six authors (members of the Neonatal Vascular Access Group of the Italian Society of Neonatology) who are experts in this field reviewed a previously prepared questionnaire (VD, GB). Consensus on a final version of the survey was achieved after three rounds of expert consultation, during which appropriate modifications to the questionnaire were proposed. The Commission for National and International Fact-Finding Investigations of the SIN ultimately approved the survey. All questions were loaded onto the SurveyMonkey platform, a complimentary tool for creating online survey forms, and underwent proofreading.

The questionnaire required approximately 15 to 20 min to complete. A cover letter containing a hyperlink to the survey was distributed by the SIN to the 114 Italian Neonatal Units. On behalf of each NICU, we requested that the survey be compiled by one attending neonatologist with specific expertise in neonatal vascular access. To ensure that only one response per center was recorded, a unique link was assigned to each institution. The survey comprised various question types, including dichotomous, multiple-choice, and open-ended response options. The survey questionnaire is available in the Supplementary File.

The survey results were reported in accordance with the checklist for reporting results of internet e-surveys (CHERRIES) [9]. No financial incentives were offered for participating in the survey. Survey Monkey Forms automatically converted every questionnaire into Excel files (Microsoft, Seattle, WA). Questionnaires underwent a thorough review for potential inconsistencies and missing data throughout the conversion process. If missing data exceeded 50%, the questionnaires were excluded from the analysis.

## Results

The overall survey response rate was 85%, with 97 responding centers (RCs) out of 114. The geographical distribution of the RCs is provided in the supplemental file. Two centers were excluded due to more than 50% incomplete data. The participating RCs collectively account for 844 beds, with a median of 8 beds per center. Ninety centers (95%) reported adhering to an institutional protocol for catheter insertion in the NICU, while 10% do not follow a specific protocol for skin disinfection prior to venous catheter insertion.

Sixty-five percent (65%) of centers use a 2% chlorhexidine solution in a 70% alcohol solution for skin disinfection prior to central catheter insertion in neonates. Ten percent (10%) utilize povidone-iodine, while two percent (2%) opt for sodium hypochlorite. Table 1 summarizes the findings by catheter type. Furthermore, ninety percent (90%) of centers utilize the same product for skin disinfection during the care of the exit site after catheter placement. Among the centers that use alcoholic chlorhexidine, sixty-five percent (65%) do so without any restrictions, thirty percent (30%) impose restrictions based on gestational age, and five percent (5%) restrict use based on neonatal weight.

**Table 1:** Use of skin disinfection based on the type of catheter to be inserted

	UVCs	ECCs	CICCs/FICCs
2% CHG in 70% alcoholic solution	56.84	58.95	64.63
10% Povidone iodine	10.53	9.47	10.98
2% CHG in aqueous solution	8.42	10.53	6.1
o.5% CHG in aqueous solution	12.63	9.47	8.54
o.5% CHG in alcoholic solution	7.37	7.37	7.32
Sodium hypochlorite	2.11	2.11	1.22

*UVCs* Umbilical venous catheters, *ECCs* Epicutaneo-Cava Catheters, *CICCs* Centrally Inserted Central Catether, *FICCs* Femoral Inserted Central Catether

Seventy-one percent (71%) of RCs use single-dose dispensers with a known amount of antiseptic solution, and all RCs consider the quantity of disinfectant to be a critical factor in the disinfection procedure. No RC that uses CHG on a daily bases scrubs the skin during disinfection.

Twenty-one percent (21%) of RCs do not remove chlorhexidine after application, whereas thirty-seven (37%) remove it due to concerns about potential skin injuries. Only one RC reported the presence of a specialized team within the NICU responsible for the management and placement of catheters within the unit.

## Discussion

The Italian survey highlighted that 65% of Italian NICU use 2% CHG in 70% IPA according to the guidelines for skin disinfection for catheter insertion [4, 10]. The primary concerns regarding its use in preterm infant stem from two factors: skin lesions caused by the quantity used and toxicity related to systemic absorption [11, 12]. Other surveys conducted in neonatal units across different geographical locations have revealed that 10% povidone iodine and 2% chlorhexidine in aqueous or alcoholic solutions are the most commonly used antiseptics for skin preparation, with varying percentages of the different antiseptic solution [13–15].

In a recent survey conducted involving 54 UK neonatal intensive care units, it was discovered that 39% of these units used chlorhexidine (CHG) either alone or in combination with alcohol. Conversely, 61% of the units opted for lower CHG concentrations (0.05–1%) or CHG without iodophor (IPA). Furthermore, 7% of units utilized sterile water or 0.9% saline solutions for extremely preterm neonates. Additionally, 11% of centers reported using CHG solutions diluted with sterile water at the bedside. 2% CHG in IPA 70% is largely recommended based on the evidence from many studies on adult and paediatric populations. The 2011 guidelines for the prevention of intravascular catheter-related infections recommend the use of at least 0.5% chlorhexidine in alcoholic solution [16]. Additionally, they recommend employing 2% CHG-70% IPA for skin antisepsis in adults and older children. Chlorhexidine exhibits excellent antiseptic and antimicrobial properties, while alcohol possesses broad-spectrum antimicrobial activity against most bacteria, fungi, and viruses (although it lacks sporicidal properties). Notably, despite CHG’s proven efficacy in neonates, the current guidelines do not recommend CHG antisepsis for infants under 2 months due to insufficient safety data in this population [17].

An ideal antiseptic should have rapid and effective action, as well as sufficient residual activity. This persistence of the antimicrobial effect suppresses the regrowth of the skin flora not removed during preparation and transient microorganisms that come into contact with the prepared site.

However, data on the efficacy and safety of various antiseptic solutions in the neonatal population are still limited. Malathi et al. compared skin bacterial clearance using 0.5% chlorhexidine in 70% isopropyl alcohol and 10% povidone iodine for intravenous cannulation in preterm neonates. Although the reduction in colony counts varied with the duration and frequency of skin cleansing, no difference was reported between the two groups [18]. Nuntnarumit et al. compared the efficacy of 1% aqueous chlorhexidine and povidone Iodine antisepsis on blood culture contamination rates in neonates weighing > 1500 g, reporting better outcomes with chlorhexidine and no adverse skin reactions with either [19]. Garland et al. found that peripheral intravenous catheter colonization reduction was better with 0.5% CHG-IPA compared to 10% povidone iodine [20]. In a randomized trial by Kieran et al., 2% chlorhexidine-70% isopropyl alcohol was compared to 10% povidone-iodine for cleaning before central line insertion in preterm infants. No adverse skin reactions were reported in either group [3]. However, the proportion of neonates with elevated TSH values was significantly higher in the povidone iodine group, with two-thirds requiring thyroxine. Jagalasar's study demonstrated that 2% CHG-IPA had better efficacy than povidone iodine for skin antisepsis in term neonates, with no significant changes in skin integrity observed across all groups [21].

Clarke et al. [22], in their ARCTIC randomized controlled feasibility trial, found no major antiseptic-related skin injuries in preterm neonates born before 34 weeks of gestation, comparing 2% chlorhexidine gluconate aqueous versus 2% chlorhexidine gluconate in 70% isopropyl alcohol for skin disinfection prior to percutaneous central venous catheterization.

The results of the present survey are consistent with European data, showing that chlorhexidine in alcoholic solution is the most used antiseptic preparation, followed by povidone-iodine.

Thirty-seven percent of RCs remove chlorhexidine due to concerns about skin lesions (chemical burns). This practice can prevent redness or skin lesions, especially in neonates with low gestational age [4, 10].

A crucial aspect in ensuring the safety of using 2% CHG in IPA 70% involves the amount of antiseptic solution used. A specific quantity is necessary for a certain surface area to minimize the risks of skin injury and overdose. This can be achieved safely only with the use of a single sterile applicator. Interestingly, our survey revealed that 100% of RCs prioritize the correct quantity to reduce the risk of skin lesions or overdoses. Moreover, seventy-one percent of RCs use 2% CHG in 70% IPA with a single pre-dosed applicator.

None of the RC scrubs the skin to avoid damaging the thin or absent stratum corneum, which could allow deeper penetration of the antiseptic but increasing the risk of lesions [11, 23].

An interesting finding from the Italian survey is that only one RC has a dedicated PICC team for neonatal vascular access. It is well known that insertion, maintenance, and complication resolution procedures are better when performed by a specialized reference team [24–27]

## Conclusions

The Italian survey revealed a steady increase in the use of CHG. Despite concerns about skin lesions, product toxicity, or efficacy, CHG remains widely utilized. A few well-defined practices can help minimize risk of complications related to the use of 2% CHG in IPA 70% in preterm infants (Table 2). The Neonatal Vascular Access Group (AVN GdS) of the Italian Society of Neonatology strongly advocates for 2% CHG in 70% IPA as the skin antiseptic of choice for any neonatal vascular access. The inclusion of an appropriate antiseptic solution (2% CHG in 70% IPA) in the insertion and maintenance bundles for neonatal vascular access [28–30], may represent a critical step toward achieving the ambitious goal of 'targeting zero' catheter related/associated infections.

**Table 2:** The practice of skin disinfection with 2% chlorhexidine gluconate (CHG) in 70% isopropyl alcohol prior to central venous catheterization in infants

Correct amounts for the area to be disinfected
Single sterile applicator
No pooling
Rewash with sterile saline
No scrubbing

## Supplementary Information

Supplementary Material 1.

Supplementary Material 2.

## Data Availability

The datasets used and analyzed during the current study is available from the corresponding author upon reasonable request.

## Abbreviations

AVN GdS: Neonatal Vascular Access Group
AWHONN: Association of Women’s Health Obstetric and Neonatal Nurses
CDC: Disease Control and Prevention
CHG: Chlorhexidine gluconate
CICCs: Centrally Inserted Central Catether
CLABSI: Central line bloodstream infections
ECCs: Epicutaneo-Cava Catheters
FDA: The Food and Drug Administration
FICCs: Femoral Inserted Central Catether
IPA: Isopropyl Alcohol
NICU: Neonatal Intensive Care Unit
RCs: Responding centers
UVCs: Umbilical venous catheters
