# Supplementary material for: The use of 2% chlorhexidine gluconate in 70% isopropyl alcohol for skin disinfection prior to central venous catheterization in infants: a national survey of the Italian Society of Neonatology
Source: Ital J Pediatr. 2025 Jun 6;51:172. doi: 10.1186/s13052-025-02016-5 (PMC12144814; doi:10.1186/s13052-025-02016-5)
Supplement: Supplementary file 1 — Supplementary Material 1. [file 13052_2025_2016_MOESM1_ESM.pdf]

Survey

1. How many intensive care beds are accredited in your center?

2. Do you use standardized recommendations (departmental or institutional) for central venous catheters in NICU?

   a. Yes

   b. No

3. Do you use a standardized protocol for skin disinfection during the placement of central venous catheters in NICU?

   a. Yes

   b. No

4. What product do you use for disinfection during the placement of umbilical venous catheters?

   a. Povidone-iodine

   b. Povidone-iodine with alcohol (before, after, or mixed)

   c. Chlorhexidine 0.5% in aqueous solution

   d. Chlorhexidine 2% in aqueous solution

   e. Chlorhexidine gluconate 2% in alcohol solution

   f. Sodium hypochlorite

   g. 0.5% CHG in alcoholic solution

   h. Other (specify)

5. What product do you use for disinfection during the placement of epicutaneo-caval catheters?

   a. Povidone-iodine

   b. Povidone-iodine with alcohol (before, after, or mixed)

   c. Chlorhexidine 0.5% in aqueous solution

   d. Chlorhexidine 2% in aqueous solution

   e. Chlorhexidine gluconate 2% in alcohol solution

   f. Sodium hypochlorite

   g. 0.5% CHG in alcoholic solution

   h. Other (specify)

6. What product do you use for disinfection during the placement of ultrasound-guided venous catheters (CICC and FICC)?

   a. Povidone-iodine

   b. Povidone-iodine with alcohol (before, after, or mixed)

   c. Chlorhexidine 0.5% in aqueous solution

   d. Chlorhexidine 2% in aqueous solution

   e. Chlorhexidine gluconate 2% in alcohol solution

   f. Sodium hypochlorite

   g. 0.5% CHG in alcoholic solution

   h. Other (specify)

7. Who decides whether to use chlorhexidine for specific patients and central venous catheter placement procedures?

   a. Neonatologist

   b. Nursing staff

   c. PICC team

d. Department protocol

e. Other (specify)

8. On what neonatal characteristics do you base the use of chlorhexidine in alcohol solution?

a. Gestational age

b. Neonatal weight

c. Postnatal age

d. No specific characteristics

e. Other (specify)

9. Do you limit the use of chlorhexidine in alcohol solution for neonates of a certain gestational age?

a. We do not use chlorhexidine in alcohol solution

b. Neonates > 24 weeks of gestational age

c. Neonates > 30 weeks of gestational age

d. Neonates > 32 weeks of gestational age

e. No gestational age limit

f. Other (specify)

10. Do you limit the use of chlorhexidine in alcohol solution for neonates of a certain post-conceptional age?

    a. We do not use chlorhexidine in alcohol solution

    b. Only in neonates of 2 weeks of age

    c. Only in neonates of 4 weeks of age

    d. No age limit

    e. Other (specify)

11. How much chlorhexidine in alcohol solution do you use for skin disinfection?

    a. Just a small amount

    b. Depends on the area to disinfect

    c. The quantity is not a concern

    d. We use a sterile single-dose applicator

12. Do you routinely remove chlorhexidine in alcohol solution from the skin after application?

    a. Yes, with saline solution

    b. Yes, with sterile saline solution

    c. No

    d. Depends on the neonate

13. If you remove chlorhexidine in alcohol solution from the skin, what is the reason?

a. It is required by procedural protocol

b. Concern for associated injuries (observed in the unit)

c. Concern for associated injuries (not observed in the unit)

d. Based on recent publications

e. Other (specify)

14. If you use a sterile single-dose applicator, how is it applied to the neonate's skin?

a. Circular movements

b. Back-and-forth lateral movement

c. Scrubbing

d. Dabbing
