# Supplementary figures and images for: The use of 2% chlorhexidine gluconate in 70% isopropyl alcohol for skin disinfection prior to central venous catheterization in infants: a national survey of the Italian Society of Neonatology
Source: Ital J Pediatr. 2025 Jun 6;51:172. doi: 10.1186/s13052-025-02016-5 (PMC12144814; doi:10.1186/s13052-025-02016-5)

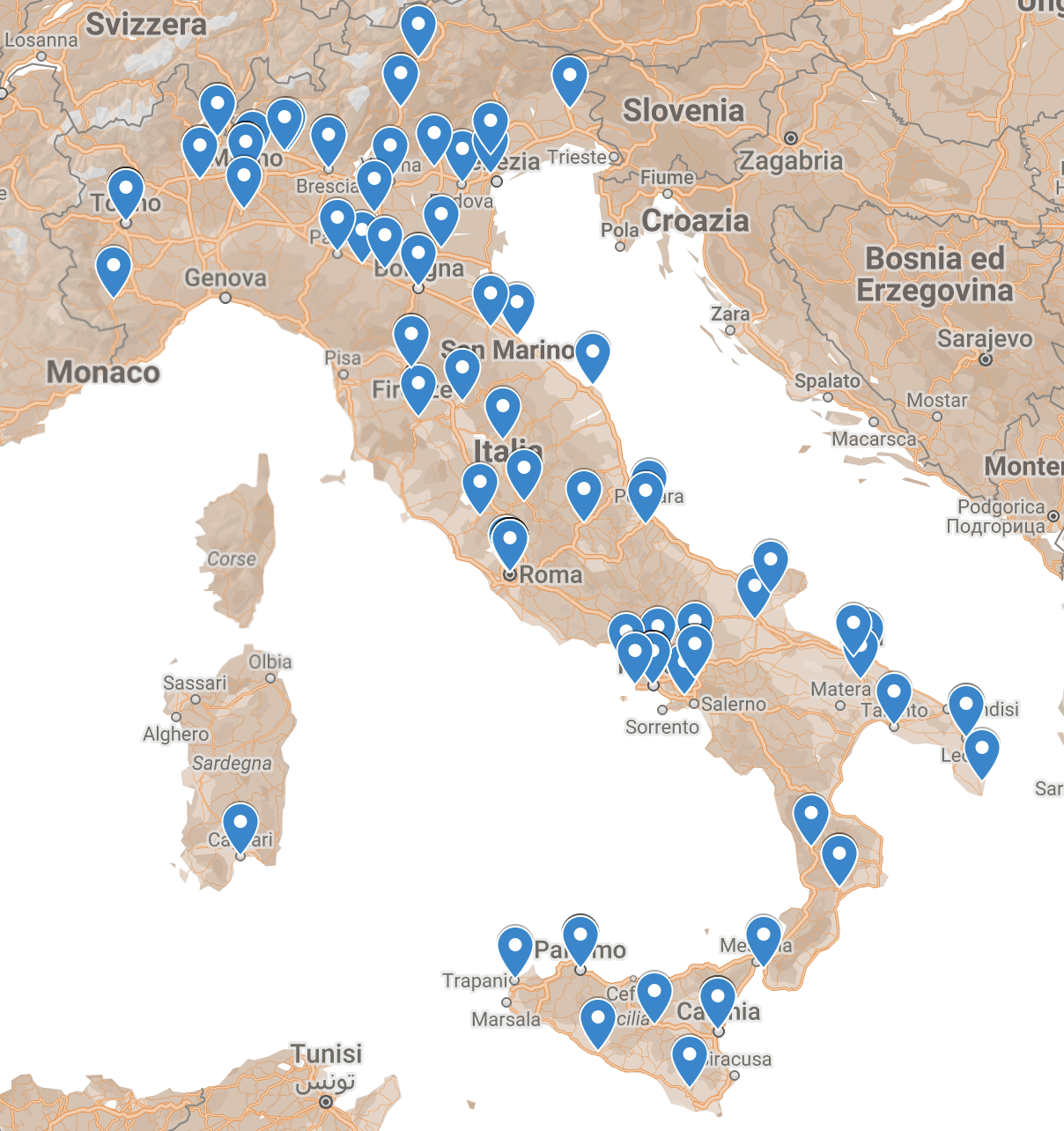

Supplement: Supplementary file 2 — Supplementary Material 2. [file 13052_2025_2016_MOESM2_ESM.png]
